# Data on lateral photocurrent along a Cu(In,Ga)Se_2_ thin film as a function of air exposure time

**DOI:** 10.1016/j.dib.2019.104668

**Published:** 2019-10-16

**Authors:** Jiseong Jang, Sangyeob Lee, Choong-Heui Chung

**Affiliations:** a Department of Materials and Manufacturing Engineering, Hanbat National University, Daejeon, 34158, Republic of Korea; b Department of Materials Science and Engineering, Hanbat National University, Daejeon, 34158, Republic of Korea

**Keywords:** Lateral photocurrent, Minority carrier diffusion length, Back-surface recombination velocity, Solar cells, CIGS

## Abstract

Wavelength-dependent (i.e. penetration-depth-dependent) lateral photocurrent (*i*_*LP*_) measurement has been used to extract depth-resolved *L*_*c*_ profiles, where *L*_*c*_ is the minority carrier collection length by diffusion. The extracted *L*_*c*_ depth-profiles can be used to determine the minority carrier diffusion length and back-surface recombination velocity in Cu(In,Ga)Se_2_ (CIGS) thin film solar cells (Chung, 2019). During the measurement of *i*_*LP*_, the CIGS thin film is generally exposed to air. The CIGS thin films can be degraded by air exposure (Metzger et al., 2009). Therefore, it will be helpful to know the effect of air exposure time of CIGS thin films on the *i*_*LP*_ values to properly estimate the electrical quality of CIGS thin films.

**Table:** Specifications Table

Subject	Electrical engineering
Specific subject area	Solar cells
Type of data	Table and Figures
How data were acquired	Keithley 2401 source meter under light illumination, a UV-vis spectrometer, and an optical power meter.
Data format	Raw, and analyzed
Parameters for data collection	Wavelength of incident light, and duration of air exposure of a Cu(In,Ga)Se_2_ thin films
Description of data collection	The lateral photocurrent values were measured from a short-circuit conditioned device, with a structure of Al/Ni/CdS/Cu(In,Ga)Se_2_/Mo under various wavelength of incident light. Al/Ni/CdS is disk-shaped, and Cu(In,Ga)Se_2_/Mo is planar stacked. The current density-voltage curve of a Cu(In,Ga)Se_2_ solar cell with a planar structure of ZnO:Al/i-ZnO/CdS/Cu(In,Ga)Se_2_/Mo was measured.
Data source location	Hanbat National University, Daejeon 34158, Republic of Korea
Data accessibility	Raw data related to Fig. 2, Fig. 3, Fig. 4 are available within this article as a supplementary file.
Related research article	Author’s name: Choong-Heui Chung Title: Method to determine the recombination characteristics of minority carriers in graded-band-gap solar cells Journal: Physical Review Applied DOI:10.1103/PhysRevApplied.12.024060

**Table:** 

**Value of the Data** • Lateral photocurrent in a custom-designed Cu(In,Ga)Se_2_ (CIGS) test pattern steadily decreased with increasing the air exposure time of the CIGS thin films. • When researchers measure the electrical quality of CIGS thin films, the thin films are typically exposed to air. The data could guide researchers to set the maximum allowed air exposure duration before measuring the electrical properties of the thin films because the thin films can be degraded by air exposure. • Lateral photocurrent along a light absorber layer as function of exposure time to a certain environment could help researchers to decide optimum storing time and an environment of the layer during fabricating solar cells.

## 1 Data

Lateral photocurrent (*i_LP_*) along a CIGS thin film has been used to determine the minority carrier diffusion length and back-surface recombination velocity in a CIGS thin film solar cell [1]. The CIGS thin film has been known to be degraded by air exposure [2]. This section is divided by two parts. One is about a CIGS test pattern, which can be used to measure *i*_*LP*_ values as a function of air exposure time, and the other is about a real CIGS solar cell. Raw data related to Figs. 2–4 are available within this article as a supplementary file.

### 1.1 CIGS test pattern

#### 1.1.1 Narrow bandwidth light

**Fig. 1:**
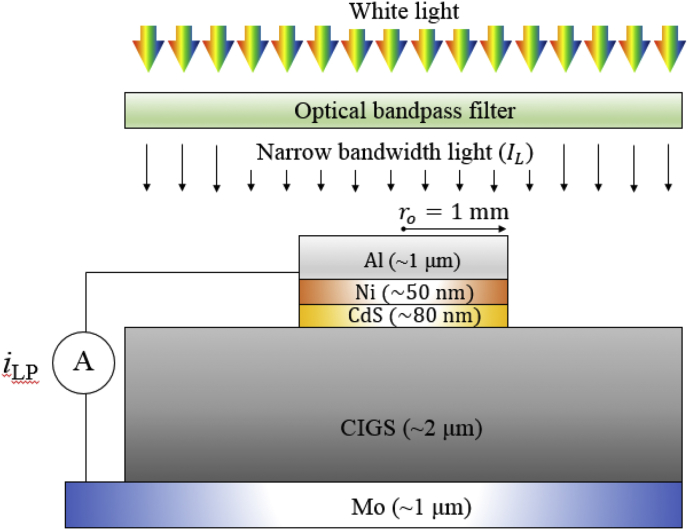
A schematic of the measurement of the lateral photocurrent in the CIGS test pattern under illumination of narrow bandwidth light with an intensity of *I*_*L*_.

**Fig. 2:**
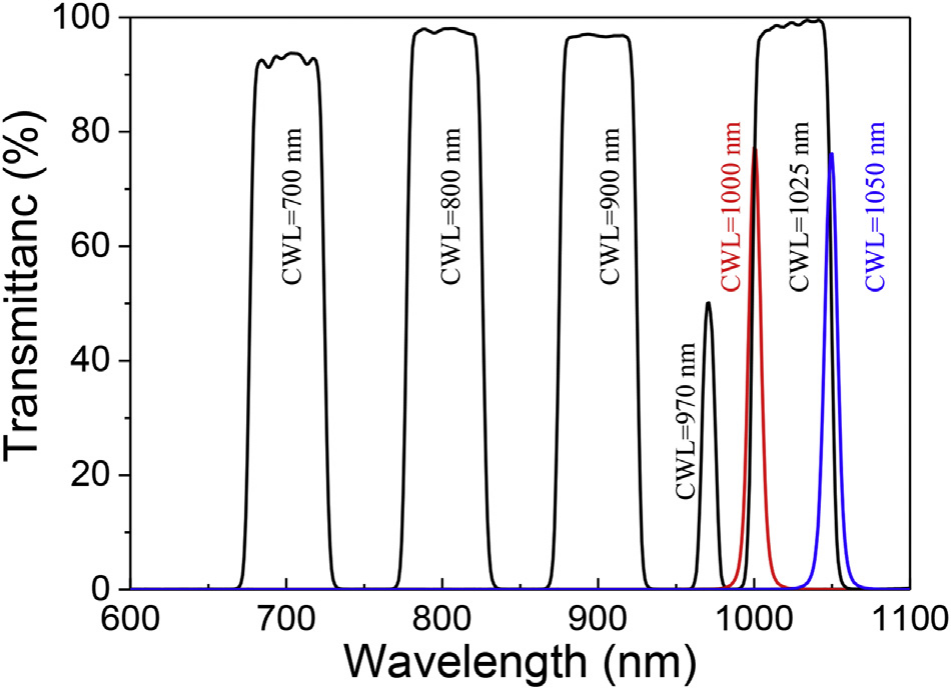
Optical transmittances of the employed seven optical bandpass filters.

In order to measure wavelength-dependent *i*_*LP*_ values in a CIGS test pattern (Fig. 1), we placed an optical bandpass filter between the sample and a white-light source to shine narrow bandwidth light on the sample. We used seven optical bandpass filters, whose optical transmittances are shown in Fig. 2. The center wavelengths (CWL), the full width half maximum (FWHM), and catalog numbers of the optical bandpass filters are summarized in Table 1. The intensities (*I*_*L*_) of narrow bandwidth light, generated by passing white-light through the optical bandpass filters, are also displayed in Table 1.

**Table 1:** Center wavelength (CWL), full width half maximum (FWHM), and catalog number of the employed optical bandpass filters. The intensities (*I*_*L*_) of narrow bandwidth light, generated by passing white-light through the optical bandpass filters.

Optical bandpass filter			Narrow bandwidth light intensity I_L_ (W/m^2^)
CWL (nm)	FWHM (nm)	Catalog number (Edmond optics)	
700	50	#84-775	1.26 × 10^2^
800	50	#84-777	9.92 × 10^1^
900	50	#84-779	1.63 × 10^2^
970	10	#67-797	6.69 × 10^0^
1000	10	#65-768	1.51 × 10^1^
1025	50	#87-860	4.48 × 10^1^
1050	10	#65-769	6.88 × 10^0^

#### 1.1.2 Lateral photocurrent

**Fig. 3:**
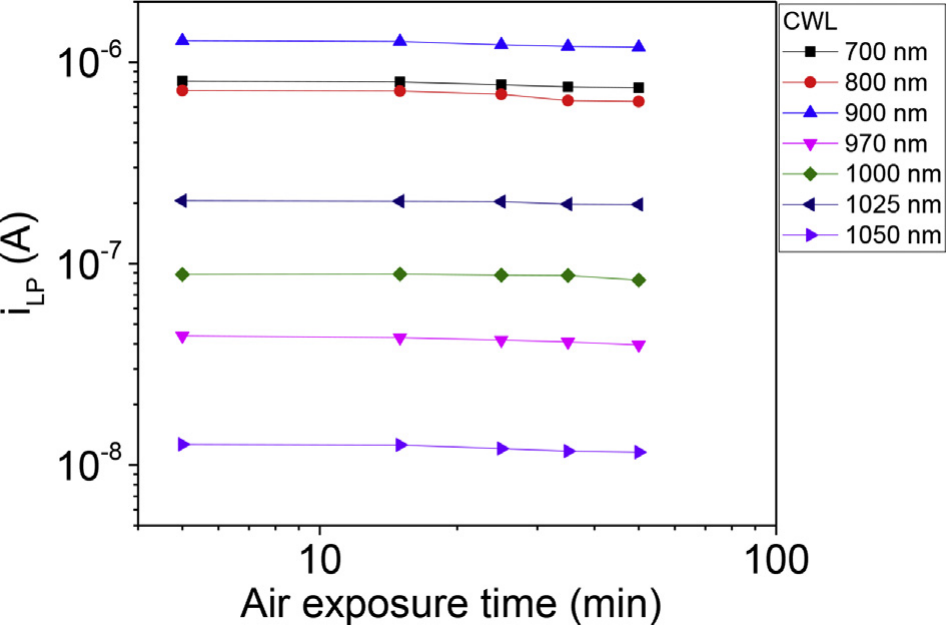
The lateral photocurrent (*i*_*LP*_) values measured in the CIGS test pattern as a function of air exposure time of the CIGS thin film.

Fig. 3 shows the *i*_*LP*_ values as a function of air exposure time of the CIGS film. The first set of *i*_*LP*_ measurement was done within 5 mins after CdS wet-etching. We set these values a reference to investigate the effect of air exposure time of the CIGS thin film on the wavelength-dependent *i*_*LP*_ values. After additional air exposure for 10 mins (total air exposure time = 15 mins), the *i*_*LP*_ values were dropped by less than 1%. With further increasing air exposure time, the *i*_*LP*_ values were steadily decreased. Upon additional air exposure for approximately 45 mins (total air exposure time = 50 mins), the *i*_*LP*_ values were dropped by 5–12%.

### 1.2 CIGS solar cell

**Fig. 4:**
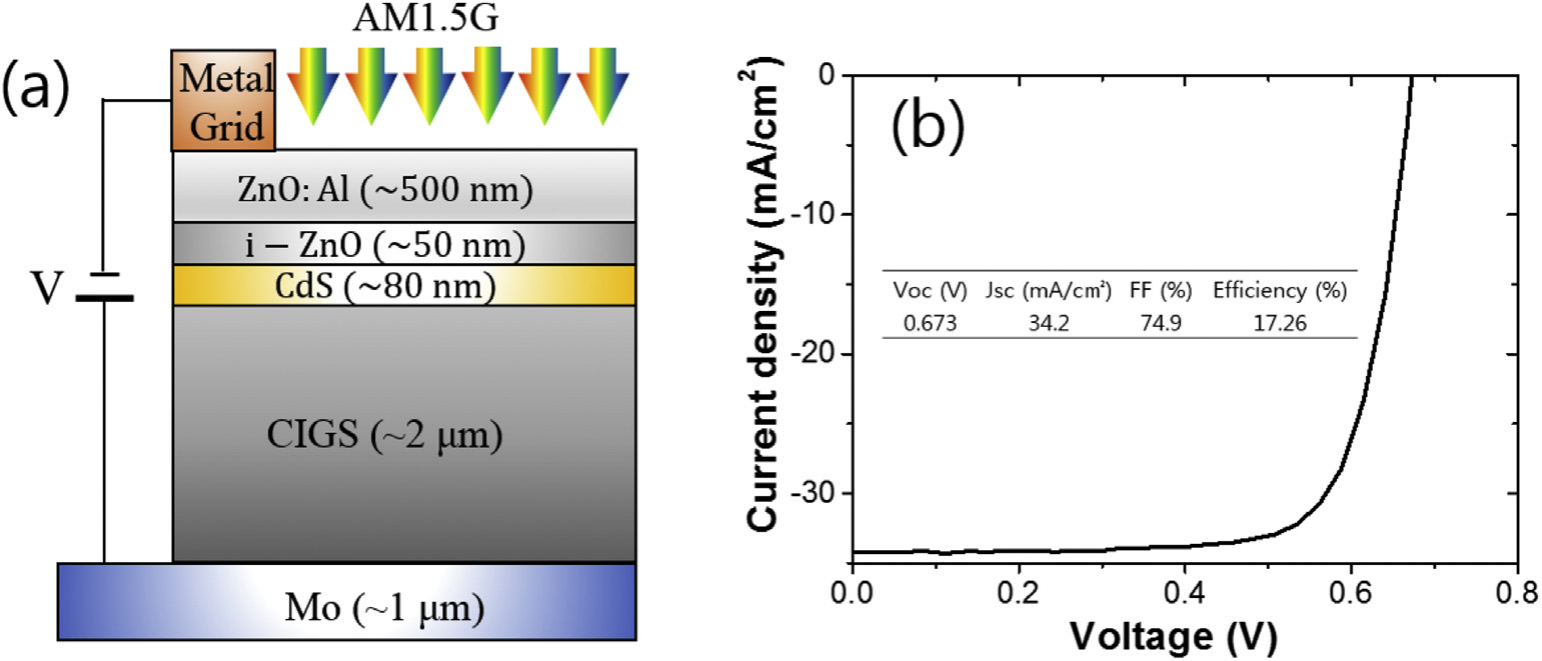
(a) A schematic of the current-voltage measurement of the fabricated CIGS solar cell under illumination of AM1.5G light. (b) The current density-voltage curve of the device, which shows an efficiency of 17.26%, an open-circuit voltage (V_oc_) of 0.673 V, a short-circuit current density (J_sc_) of 34.2 mA/cm^2^, and a fill factor (FF) of 74.9%.

The electrical quality of the employed CIGS layer can be evaluated by characterizing a real CIGS solar cell, where the whole surface of the CIGS thin film is covered by CdS. The current density-voltage curve of the fabricated CIGS solar cell, whose schematic is shown in Fig. 4(a). The device shows an efficiency of 17.26%, an open-circuit voltage (V_oc_) of 0.673 V, a short-circuit current density (J_sc_) of 34.2 mA/cm^2^, and a fill factor (FF) of 74.9%.

## 2 Experimental design, materials, and methods

This section is divided by two parts. The first one is the fabrication and characterization method for a CIGS test pattern, and the other one is the fabrication and characterization method of a real CIGS solar cell.

### 2.1 CIGS test pattern

#### 2.1.1 Fabrication

A CIGS test pattern with a structure of Al/Ni/CdS/CIGS/Mo was custom-designed to measure the *i*_*LP*_ values along the CIGS thin film (Fig. 1). Al/Ni/CdS is disk-shaped with a radius (*r*_*o*_) of 1 mm, and CIGS/Mo is planar stacked. Mo was prepared by DC-sputtering on a soda-lime glass, CIGS layer by three stage co-evaporation, CdS by chemical bath deposition, and Al/Ni by e-beam evaporation. More details on the preparation methods can be found elsewhere [3,4]. Mo, CIGS, and CdS were planar stacked on the substrate, and Al/Ni were deposited through a shadow mask with a disk-shaped hole. The CdS layer was removed using wet chemical just before starting to measure the *i*_*LP*_ values.

#### 2.1.2 Characterization

Here, we describe the measurement of components which are necessary to measure the wavelength-dependent *i*_*LP*_ values in the CIGS test pattern (Fig. 1). A white-light was generated by a solar simulator (Model 11002 SunLite, Abet technologies). Narrow bandwidth light was generated by passing white-light through an optical bandpass filter. Wavelength of incident light can be modulated by placing an appropriate optical bandpass filter between the sample and the white-light source. The optical transmittance of the optical bandpass filters were measure by a UV–Visible spectrometer (Genesys 10S, Thermo scientific). The intensities of the narrow bandwith light were measured using an optical power meter (S120VC, PM100USB, Thorlabs). The *i*_*LP*_ values were measured from a short-circuit condition between Al and Mo (Fig. 1).

### 2.2 Fabrication and characterization of a CIGS solar cell

A CIGS solar cell has a planar structure of ZnO:Al/i-ZnO/CdS/CIGS/Mo (Fig. 4(a)). The preparation methods for Mo, CIGS, and CdS is same to the case of fabrication of above test structure. Double-layered ZnO:Al/i-ZnO transparent conducting oxides were prepared by radio-frequency sputtering. The current-voltage curve of the CIGS solar cell was measured using Keithley 2401 source meter under AM1.5G light illumination. The white-light was generated by a solar simulator (Model 11002 SunLite, Abet technologies). The light intensity shining on the device was controlled by adjusting the distance between the light source and the device. The distance was set for a reference cell (15151-KG5, Abet technologies) to produce a voltage of 100 mV for the light spectrum to be AM1.5G.
