# Supplementary material for: Data on lateral photocurrent along a Cu(In,Ga)Se2 thin film as a function of air exposure time
Source: Data Brief. 2019 Oct 16;27:104668. doi: 10.1016/j.dib.2019.104668 (PMC6831709; doi:10.1016/j.dib.2019.104668)
Supplement: Multimedia component 1 [file mmc1.pdf]

Fig. 2

Wavelength (nm)	Transmittance (%)						
	CWL (nm)/FWHM (nm)						
	700/50	800/50	900/50	970/10	1000/10	1025/50	1050/10
600	0.0112	0.0076	0.0084	-0.0096	-0.005	0.0169	-0.0023
601	0.0094	0.0085	0.0091	-0.0087	-0.0056	0.0185	-0.0022
602	0.0095	0.0093	0.009	-0.0103	-0.0058	0.0182	-0.0052
603	0.0082	0.0073	0.0106	-0.0111	-0.0052	0.0171	-0.0046
604	0.0086	0.0099	0.0117	-0.009	-0.0067	0.017	-0.004
605	0.0068	0.0114	0.0097	-0.0095	-0.0048	0.0175	-0.0034
606	0.009	0.0114	0.0095	-0.0097	-0.0046	0.0163	-0.0053
607	0.0078	0.0135	0.0099	-0.0095	-0.0043	0.0169	-0.0028
608	0.0091	0.014	0.0117	-0.0105	-0.0058	0.0135	-0.0037
609	0.0101	0.015	0.0091	-0.0094	-0.0047	0.0152	-0.0033
610	0.0089	0.0151	0.0095	-0.0093	-0.0057	0.0153	-0.0039
611	0.0082	0.0146	0.0079	-0.0105	-0.0051	0.0191	-0.0026
612	0.0082	0.0129	0.009	-0.0102	-0.0048	0.0171	-0.0039
613	0.0088	0.0096	0.0083	-0.01	-0.0053	0.0174	-0.0024
614	0.0075	0.0091	0.0082	-0.0108	-0.0046	0.0169	-0.0043
615	0.0086	0.008	0.0107	-0.0085	-0.0066	0.016	-0.0033
616	0.0093	0.0075	0.0115	-0.01	-0.007	0.0166	-0.0021
617	0.0097	0.0089	0.0106	-0.0104	-0.0043	0.0163	-0.0049
618	0.008	0.0059	0.0094	-0.0101	-0.0043	0.0164	-0.0038
619	0.0105	0.0052	0.0111	-0.0096	-0.0045	0.016	-0.0027
620	0.0085	0.0047	0.0094	-0.0095	-0.0047	0.0139	-0.0033
621	0.0095	0.0053	0.0093	-0.009	-0.0054	0.0136	-0.0035
622	0.0088	0.0062	0.0096	-0.0079	-0.0042	0.0159	-0.003
623	0.0104	0.0064	0.0119	-0.0095	-0.0048	0.0153	-0.0057
624	0.0113	0.0068	0.0092	-0.0088	-0.0032	0.0151	-0.0035
625	0.0095	0.0077	0.0117	-0.0085	-0.0053	0.0173	-0.0045
626	0.0108	0.0095	0.0108	-0.0091	-0.004	0.0158	-0.0028
627	0.0101	0.008	0.0103	-0.009	-0.0043	0.0161	-0.0045
628	0.0118	0.0115	0.0097	-0.0087	-0.0046	0.0165	-0.0041
629	0.0097	0.0108	0.0095	-0.0095	-0.0051	0.018	-0.0024
630	0.0127	0.0101	0.0093	-0.0112	-0.0049	0.016	-0.0047
631	0.0106	0.0118	0.0103	-0.0103	-0.0054	0.0185	-0.0032
632	0.0111	0.0148	0.0094	-0.0093	-0.0031	0.02	-0.003
633	0.0128	0.0149	0.0113	-0.0103	-0.0038	0.0244	-0.0033
634	0.0116	0.0178	0.0114	-0.0084	-0.0052	0.0217	-0.0021
635	0.012	0.0178	0.0098	-0.0086	-0.0035	0.0233	-0.0037
636	0.0121	0.0169	0.0094	-0.0093	-0.0035	0.0239	-0.0034
637	0.0137	0.0154	0.0095	-0.0084	-0.004	0.0191	-0.0033
638	0.0143	0.0143	0.0093	-0.0102	-0.0052	0.0183	-0.0036
639	0.0146	0.0107	0.0112	-0.0089	-0.0055	0.0161	-0.0036

640	0.0146	0.0123	0.0089	-0.0089	-0.0049	0.0155	-0.0036
641	0.0151	0.0121	0.0099	-0.0089	-0.0048	0.015	-0.0031
642	0.0157	0.0139	0.0102	-0.0089	-0.0049	0.0142	-0.0032
643	0.0176	0.0159	0.0103	-0.008	-0.0036	0.0146	-0.0036
644	0.0153	0.0178	0.0066	-0.0083	-0.0042	0.0145	-0.0027
645	0.0159	0.0202	0.0089	-0.0084	-0.0035	0.0153	-0.0037
646	0.0161	0.0241	0.0097	-0.0092	-0.0033	0.0143	-0.0015
647	0.0144	0.0268	0.0055	-0.0079	-0.0042	0.0146	-0.0038
648	0.018	0.0279	0.0082	-0.0079	-0.0044	0.0159	-0.0031
649	0.0205	0.0279	0.0094	-0.007	-0.0044	0.0152	-0.0024
650	0.0193	0.026	0.0087	-0.0092	-0.0038	0.0156	-0.0039
651	0.0204	0.0218	0.007	-0.0101	-0.0052	0.0173	-0.0036
652	0.0231	0.0194	0.0084	-0.0093	-0.0038	0.0175	-0.0055
653	0.0246	0.0179	0.0082	-0.0081	-0.0047	0.0207	-0.0029
654	0.0241	0.0137	0.0079	-0.0076	-0.0043	0.021	-0.0014
655	0.028	0.0099	0.0067	-0.0084	-0.0043	0.0215	-0.0013
656	0.03	0.0102	0.0056	-0.0077	-0.0012	0.0189	-0.0016
657	0.0338	0.0083	0.0093	-0.0072	-0.0029	0.0183	-0.0012
658	0.0352	0.0094	0.0104	-0.0056	-0.0021	0.0151	-0.0021
659	0.0409	0.0106	0.0084	-0.0062	-0.0024	0.0152	-0.0014
660	0.0438	0.0114	0.0073	-0.0067	-0.0024	0.0143	-0.0015
661	0.05	0.0106	0.0089	-0.0059	-0.0038	0.0154	-7.00E-04
662	0.0513	0.0089	0.0084	-0.0061	-0.0028	0.0135	-0.0011
663	0.0616	0.0123	0.0084	-0.0065	-0.0025	0.0133	-0.002
664	0.0692	0.0129	0.0077	-0.0067	-0.0014	0.0134	-0.002
665	0.0826	0.0132	0.0085	-0.0065	-0.0025	0.0136	-0.0015
666	0.1007	0.0127	0.0066	-0.0059	-0.0019	0.0116	-8.00E-04
667	0.1338	0.013	0.0058	-0.0068	-0.0035	0.0107	-0.0017
668	0.1918	0.0136	0.007	-0.0042	-0.0023	0.0121	-0.0016
669	0.3221	0.0107	0.008	-0.0049	-0.0031	0.0123	-0.001
670	0.5842	0.0117	0.0072	-0.0048	-0.0023	0.0113	-0.0011
671	1.1924	0.0111	0.0074	-0.0046	-0.0015	0.0107	-0.0023
672	2.5403	0.0084	0.0082	-0.0041	-0.0025	0.0136	-0.0025
673	5.7386	0.0088	0.0071	-0.0049	-0.0019	0.0136	-9.00E-04
674	12.6135	0.0066	0.0064	-0.0046	-0.0025	0.0146	-5.00E-04
675	23.1291	0.0076	0.0059	-0.005	-0.0012	0.0148	-0.0011
676	37.7264	0.0063	0.0071	-0.005	-0.0024	0.015	-0.002
677	54.3238	0.0099	0.0077	-0.0042	-0.0017	0.0136	-0.0014
678	69.6907	0.0098	0.0053	-0.0044	-0.0018	0.0152	-9.00E-04
679	80.6439	0.0098	0.0067	-0.0045	-0.0013	0.0127	-0.0012
680	87.7378	0.0111	0.006	-0.0048	-0.0018	0.0135	-5.00E-04
681	90.9595	0.0118	0.0045	-0.0043	-0.0019	0.0122	-0.0013
682	91.9484	0.0097	0.0045	-0.004	-0.0017	0.012	-7.00E-04
683	92.3407	0.0072	0.0057	-0.0042	-0.0019	0.0111	-0.0018

684	92.5609	0.0077	0.007	-0.0053	-0.0022	0.0114	-7.00E-04
685	92.4743	0.0075	0.0082	-0.005	-0.0019	0.0107	-0.0012
686	92.0919	0.0045	0.0079	-0.0039	-8.00E-04	0.0118	-0.0014
687	91.6175	0.0039	0.0072	-0.0047	-0.002	0.0107	-0.0019
688	91.2679	0.0059	0.0064	-0.0062	-0.0022	0.0124	-0.0012
689	91.2467	0.0054	0.0062	-0.0057	-0.0023	0.0108	-0.0011
690	91.5941	0.0044	0.0071	-0.0053	-0.0028	0.0127	-9.00E-04
691	92.2057	0.0031	0.0079	-0.0052	-0.0021	0.0117	-0.0024
692	92.8294	0.0055	0.0068	-0.0039	-0.0026	0.0127	-0.002
693	93.1478	0.0071	0.0071	-0.0046	-0.0022	0.0117	-0.0013
694	93.1728	0.0059	0.0051	-0.0054	-0.0024	0.0144	-0.001
695	93.0262	0.0053	0.0078	-0.0053	-0.0014	0.0114	-0.0021
696	92.8062	0.0038	0.0078	-0.0063	-0.0024	0.0134	0
697	92.7672	0.0046	0.0057	-0.0053	-0.0029	0.0117	-0.0016
698	92.8751	0.0051	0.0059	-0.0053	-0.003	0.0123	-5.00E-04
699	93.133	0.003	0.0041	-0.0051	-0.0034	0.0119	-0.002
700	93.3952	0.0042	0.0055	-0.0052	-0.0017	0.0114	-0.0012
701	93.5847	0.003	0.0053	-0.0048	-0.0021	0.012	-0.002
702	93.6866	0.0027	0.006	-0.0053	-0.0022	0.0109	-0.0023
703	93.7385	0.0044	0.0042	-0.0059	-0.002	0.0121	-0.0018
704	93.7116	0.004	0.0027	-0.006	-0.0024	0.0118	-0.0018
705	93.7065	0.0032	0.0041	-0.0069	-0.0024	0.0127	-0.0019
706	93.6902	0.0035	0.0043	-0.0052	-0.0021	0.0131	-0.0025
707	93.6648	0.0051	0.0054	-0.0061	-0.0027	0.0124	-0.0021
708	93.5839	0.0058	0.0058	-0.0067	-0.003	0.0126	-0.0011
709	93.3851	0.0037	0.0049	-0.0062	-0.0025	0.012	-0.0019
710	93.0229	0.0041	0.0062	-0.0048	-0.0027	0.0137	-0.0033
711	92.5868	0.0038	0.0058	-0.0065	-0.0021	0.0126	-8.00E-04
712	92.0758	0.0045	0.007	-0.0062	-0.0022	0.0127	-0.0022
713	91.8018	0.005	0.0066	-0.0057	-0.0022	0.0129	-0.0023
714	91.8616	0.0048	0.0055	-0.0057	-0.0022	0.0122	-9.00E-04
715	92.1797	0.0028	0.0048	-0.0067	-0.0023	0.0133	-0.0013
716	92.5607	0.0051	0.0038	-0.0058	-0.0019	0.0124	-0.0012
717	92.7922	0.0057	0.0049	-0.0051	-0.0023	0.0125	-9.00E-04
718	92.753	0.0039	0.0055	-0.0054	-0.0018	0.0134	-0.003
719	92.4371	0.0041	0.0066	-0.0057	-0.0021	0.0139	-0.0025
720	91.3425	0.0057	0.0054	-0.0054	-0.0017	0.0131	-0.0023
721	88.4995	0.0066	0.0055	-0.006	-0.0011	0.0134	-0.0011
722	82.4473	0.0042	0.0031	-0.0056	-0.0021	0.0121	-0.0022
723	71.6118	0.0055	0.0049	-0.0054	-0.0016	0.0136	-7.00E-04
724	56.6884	0.0063	0.0045	-0.0054	-0.002	0.013	-0.0022
725	40.8582	0.0061	0.006	-0.0055	-0.0022	0.0143	-0.0028
726	26.2382	0.0057	0.0071	-0.0069	-0.0018	0.013	-0.0026
727	15.2523	0.0041	0.006	-0.0067	-0.0016	0.0138	-0.0016

728	7.5424	0.0071	0.0064	-0.0065	-0.0023	0.0134	-0.001
729	3.6359	0.0067	0.0041	-0.0058	-0.0022	0.0139	-0.0013
730	1.7948	0.0051	0.0038	-0.006	-0.0028	0.0142	-0.0016
731	0.9036	0.0058	0.0053	-0.0061	-0.0025	0.0148	-0.0013
732	0.495	0.0073	0.0059	-0.007	-0.0024	0.0143	-0.0018
733	0.3001	0.0088	0.0065	-0.0058	-0.0019	0.015	-0.0027
734	0.1939	0.0062	0.0064	-0.0056	-0.002	0.0157	-0.0023
735	0.1355	0.0063	0.0056	-0.0073	-0.002	0.0153	-3.00E-04
736	0.0984	0.0081	0.004	-0.0074	-0.0012	0.0174	-0.001
737	0.0721	0.0086	0.0041	-0.0048	-0.0015	0.016	-0.0029
738	0.0582	0.007	0.0039	-0.0056	-0.0011	0.0159	-0.0011
739	0.0465	0.0075	0.0057	-0.0068	-0.0019	0.0131	-0.0013
740	0.0394	0.0104	0.0078	-0.006	0	0.0151	-0.0015
741	0.0344	0.01	0.0069	-0.0066	8.00E-04	0.0129	-9.00E-04
742	0.0287	0.0084	0.0048	-0.0064	0.0016	0.0132	-0.0031
743	0.0271	0.0098	0.0042	-0.0072	0.0034	0.0126	-0.0011
744	0.0241	0.0099	0.0049	-0.0059	0.0043	0.0148	-0.0015
745	0.0227	0.0101	0.0069	-0.0064	0.0078	0.0149	-7.00E-04
746	0.0223	0.0105	0.0057	-0.0071	0.0113	0.0178	-0.0019
747	0.0204	0.0101	0.0067	-0.007	0.0134	0.02	-9.00E-04
748	0.0221	0.0133	0.0065	-0.0073	0.0163	0.0233	-0.0014
749	0.0197	0.0136	0.0057	-0.007	0.0184	0.0285	-0.0013
750	0.0187	0.0121	0.004	-0.0062	0.0185	0.0323	-0.0022
751	0.0158	0.0127	0.0048	-0.0073	0.018	0.0353	-0.0013
752	0.0139	0.0151	0.0054	-0.0073	0.0162	0.0352	-0.0014
753	0.0142	0.0153	0.0079	-0.0063	0.0151	0.0361	-0.0011
754	0.0106	0.0156	0.007	-0.0058	0.0117	0.0324	-0.0017
755	0.0119	0.0139	0.006	-0.0063	0.0087	0.0308	-0.0016
756	0.012	0.0177	0.005	-0.0069	0.0065	0.0272	-0.0013
757	0.0118	0.0203	0.0054	-0.0051	0.0026	0.0256	-0.0023
758	0.0108	0.0201	0.0053	-0.007	4.00E-04	0.0229	-0.0012
759	0.0104	0.0199	0.0058	-0.0068	0.0012	0.0214	-0.0017
760	0.0103	0.0238	0.0055	-0.0069	-0.0013	0.0201	-0.0013
761	0.0092	0.0273	0.0067	-0.0063	-0.0017	0.0192	-5.00E-04
762	0.0103	0.0295	0.0063	-0.0084	-0.0015	0.017	-0.0023
763	0.009	0.0323	0.0061	-0.0066	-0.0031	0.0161	-0.0021
764	0.0106	0.0381	0.0041	-0.0078	-0.0034	0.0168	-0.0019
765	0.0091	0.0483	0.0033	-0.0056	-0.0032	0.0162	-0.0011
766	0.0105	0.0643	0.0059	-0.007	-0.0038	0.0162	-0.0023
767	0.0102	0.088	0.0076	-0.0066	-0.003	0.015	-0.0025
768	0.009	0.1349	0.007	-0.0067	-0.0023	0.0156	-0.0015
769	0.0107	0.2227	0.0073	-0.0072	-0.0024	0.0156	-0.0012
770	0.0067	0.3953	0.0049	-0.0057	-0.0024	0.0163	-0.003
771	0.008	0.7997	0.0058	-0.007	-0.0022	0.0159	-0.0015

772	0.0086	1.6409	0.0058	-0.0076	-0.0017	0.0182	-0.0016
773	0.0076	3.8641	0.0075	-0.0067	-0.003	0.0175	4.00E-04
774	0.0072	8.4403	0.0084	-0.0053	-0.004	0.0165	-0.0011
775	0.0075	16.7439	0.0073	-0.0069	-0.0029	0.0172	-0.0017
776	0.0089	30.3246	0.0077	-0.0073	-0.0035	0.0188	-7.00E-04
777	0.0059	46.8424	0.0053	-0.0071	-0.0025	0.017	-0.0021
778	0.0068	64.4338	0.0071	-0.0066	-0.0028	0.0167	-0.0014
779	0.0071	78.7735	0.005	-0.0075	-0.0039	0.0157	-0.0025
780	0.0069	88.8016	0.0078	-0.0065	-0.0033	0.0151	-0.0014
781	0.0078	94.6923	0.0086	-0.0057	-0.003	0.0182	-0.0019
782	0.0059	96.7419	0.0091	-0.0069	-0.0037	0.015	-0.0016
783	0.0079	97.2179	0.0053	-0.0073	-0.0021	0.0169	-0.0018
784	0.0061	97.3645	0.0055	-0.0073	-0.0038	0.0156	-0.0025
785	0.0073	97.5636	0.0054	-0.0071	-0.0033	0.0154	-0.0038
786	0.0061	97.7584	0.0066	-0.0088	-0.0045	0.0157	-0.0046
787	0.0053	97.8938	0.007	-0.009	-0.0051	0.0151	-0.0032
788	0.0065	97.9555	0.0079	-0.0082	-0.0033	0.0149	-0.0037
789	0.0039	97.942	0.0066	-0.0074	-0.0039	0.0161	-0.0021
790	0.0063	97.8639	0.006	-0.0086	-0.005	0.0146	-0.0037
791	0.0049	97.7296	0.0065	-0.0086	-0.0042	0.0157	-0.0041
792	0.0059	97.5663	0.0063	-0.0099	-0.0038	0.0136	-0.0026
793	0.0039	97.4145	0.0078	-0.0097	-0.0038	0.0144	-0.0033
794	0.0049	97.3078	0.0092	-0.0093	-0.0048	0.0136	-0.002
795	0.0079	97.2682	0.0112	-0.0091	-0.0048	0.0163	-0.0041
796	0.0054	97.3324	0.0093	-0.0067	-0.0042	0.0141	-0.0024
797	0.0069	97.463	0.0085	-0.0082	-0.0042	0.0159	-0.0023
798	0.0071	97.6272	0.0079	-0.009	-0.0041	0.014	-0.0031
799	0.0069	97.7803	0.0095	-0.0082	-0.0032	0.0148	-0.0026
800	0.0057	97.8957	0.0107	-0.0088	-0.0041	0.0168	-0.0036
801	0.0076	97.9709	0.0108	-0.0095	-0.0045	0.0148	-0.0034
802	0.0076	98.0015	0.0105	-0.0105	-0.0045	0.0147	-0.0031
803	0.0052	98.0072	0.0098	-0.0092	-0.004	0.0152	-0.0039
804	0.0083	98.0141	0.009	-0.0081	-0.0031	0.017	-0.0026
805	0.0065	98.0265	0.0104	-0.0078	-0.0045	0.0155	-0.0062
806	0.0083	98.0486	0.0117	-0.0093	-0.004	0.0156	-0.0037
807	0.0053	98.0398	0.013	-0.0079	-0.0044	0.0135	-0.0039
808	0.005	98.0085	0.0115	-0.0081	-0.005	0.0153	-0.0035
809	0.0058	97.943	0.0096	-0.0078	-0.0043	0.0141	-0.0037
810	0.0052	97.8501	0.0106	-0.0071	-0.0041	0.0162	-0.0036
811	0.0058	97.7449	0.0079	-0.0068	-0.0055	0.0141	-0.0034
812	0.0048	97.6275	0.0099	-0.0076	-0.0032	0.0152	-0.0032
813	0.0067	97.5243	0.0116	-0.0077	-0.0036	0.0137	-0.0043
814	0.0072	97.4447	0.0118	-0.0082	-0.0034	0.0169	-0.0035
815	0.0053	97.3675	0.0131	-0.0072	-0.0052	0.0149	-0.003

816	0.0073	97.2731	0.0106	-0.0069	-0.0045	0.0156	-0.0025
817	0.0057	97.1786	0.0098	-0.0088	-0.0053	0.0149	-0.003
818	0.0076	97.0955	0.0123	-0.011	-0.0052	0.0149	-0.0032
819	0.0063	97.0736	0.0132	-0.0085	-0.0049	0.017	-0.0021
820	0.0068	96.9976	0.0135	-0.0078	-0.004	0.0151	-0.0029
821	0.0066	96.4772	0.0151	-0.0088	-0.0038	0.0153	-0.0037
822	0.0053	94.4612	0.0124	-0.0097	-0.0038	0.0142	-0.003
823	0.0057	89.5536	0.0128	-0.0089	-0.0039	0.0151	-0.0038
824	0.0063	81.0867	0.0139	-0.009	-0.0043	0.0155	-0.0027
825	0.007	67.2689	0.014	-0.0072	-0.005	0.0157	-0.0038
826	0.0057	51.2461	0.0136	-0.0092	-0.0044	0.0132	-0.0037
827	0.0083	35.2061	0.0136	-0.0087	-0.0027	0.0147	-0.0046
828	0.0093	22.0969	0.0129	-0.0089	-0.0019	0.0144	-0.0031
829	0.0087	12.4691	0.0131	-0.0073	-0.0036	0.0157	-0.0041
830	0.0077	6.4638	0.0126	-0.0081	-0.003	0.0149	-0.0023
831	0.0067	3.358	0.0149	-0.0098	-0.0023	0.0147	-0.0038
832	0.006	1.7863	0.0145	-0.0096	-0.003	0.0162	-0.0024
833	0.0041	0.9605	0.0147	-0.0075	-0.0025	0.0163	-0.0039
834	0.0062	0.5455	0.016	-0.0077	-0.0014	0.015	-0.0043
835	0.006	0.3281	0.0155	-0.0101	-0.0015	0.0155	-0.002
836	0.0059	0.2049	0.0132	-0.0093	-0.0011	0.0149	-0.0031
837	0.0048	0.1361	0.0149	-0.0076	-7.00E-04	0.0163	-0.0033
838	0.0072	0.0932	0.0159	-0.0084	4.00E-04	0.0153	-0.0036
839	0.0086	0.0678	0.017	-0.0092	0.0012	0.0142	-0.0023
840	0.0063	0.0541	0.0183	-0.0073	0	0.0154	-0.002
841	0.0083	0.0425	0.018	-0.009	0.0016	0.0132	-0.0033
842	0.0059	0.0363	0.0167	-0.0083	0.003	0.0143	-0.003
843	0.0054	0.0286	0.0139	-0.0087	2.00E-04	0.0142	-0.0025
844	0.0047	0.0277	0.0176	-0.0068	0.0014	0.016	-6.00E-04
845	0.0024	0.0217	0.018	-0.0086	-5.00E-04	0.0139	-0.0016
846	0.0047	0.02	0.0192	-0.0084	-2.00E-04	0.0156	-0.0027
847	0.0022	0.0189	0.0192	-0.0089	-5.00E-04	0.0146	-0.0017
848	0.0044	0.0195	0.0189	-0.0058	0.001	0.0154	6.00E-04
849	0.0057	0.0177	0.02	-0.0062	0.001	0.0161	5.00E-04
850	0.0055	0.015	0.0201	-0.0073	0	0.0151	7.00E-04
851	0.0053	0.0128	0.0205	-0.0068	-1.00E-04	0.0161	-5.00E-04
852	0.005	0.0148	0.0235	-0.0046	-4.00E-04	0.0145	6.00E-04
853	0.006	0.0136	0.0243	-0.0063	5.00E-04	0.0159	9.00E-04
854	0.0059	0.0119	0.0249	-0.0068	1.00E-04	0.0145	-1.00E-04
855	0.0088	0.0106	0.0248	-0.0049	0	0.0144	9.00E-04
856	0.0093	0.0113	0.0259	-0.0057	-0.0013	0.0132	0.001
857	0.0145	0.0117	0.0296	-0.0054	-0.0015	0.0143	-2.00E-04
858	0.0155	0.0111	0.0329	-0.0059	-7.00E-04	0.0125	0.001
859	0.0159	0.008	0.0376	-0.0053	-0.0014	0.014	-0.0011

860	0.0135	0.0087	0.0389	-0.0066	-5.00E-04	0.0137	-3.00E-04
861	0.0075	0.0102	0.043	-0.0066	-0.0013	0.0134	-3.00E-04
862	0.0052	0.0091	0.0516	-0.006	-7.00E-04	0.012	-8.00E-04
863	0.0024	0.0073	0.0649	-0.0059	-5.00E-04	0.0132	7.00E-04
864	0.0033	0.0076	0.085	-0.0054	-0.002	0.0143	-0.0013
865	0.0019	0.01	0.121	-0.0061	-1.00E-04	0.0136	1.00E-04
866	0.0023	0.008	0.1866	-0.0062	0	0.0143	-0.0013
867	0.0027	0.0078	0.3125	-0.0067	-8.00E-04	0.0141	2.00E-04
868	0.0015	0.0072	0.5754	-0.0061	-0.0019	0.0135	-6.00E-04
869	0.0041	0.0076	1.1509	-0.0053	-0.001	0.0145	4.00E-04
870	0.0024	0.009	2.3987	-0.0055	-6.00E-04	0.0142	-1.00E-04
871	0.0051	0.0071	5.267	-0.0055	-8.00E-04	0.0137	0.0014
872	0.0032	0.006	10.8268	-0.006	-5.00E-04	0.0146	1.00E-04
873	0.0035	0.0065	20.6542	-0.0057	-8.00E-04	0.0124	3.00E-04
874	0.004	0.0091	34.1919	-0.0057	0	0.0148	7.00E-04
875	0.0034	0.0072	50.3924	-0.0058	-7.00E-04	0.012	4.00E-04
876	0.0033	0.005	67.6925	-0.0053	-4.00E-04	0.0126	9.00E-04
877	0.0025	0.0057	81.0442	-0.0053	4.00E-04	0.0108	0.001
878	0.0042	0.0064	90.0164	-0.0053	0	0.0125	5.00E-04
879	0.0015	0.0065	94.461	-0.0058	0.0011	0.012	0
880	0.0029	0.0052	95.9813	-0.0054	9.00E-04	0.014	3.00E-04
881	0.0014	0.0057	96.3568	-0.0054	0.0013	0.013	-3.00E-04
882	0.0025	0.0063	96.479	-0.0055	8.00E-04	0.012	-0.0015
883	0.0036	0.0073	96.5516	-0.0051	0.0021	0.0124	-2.00E-04
884	0.0056	0.0054	96.5934	-0.0042	0.0018	0.0131	-9.00E-04
885	0.01	0.0043	96.6304	-0.0051	0.0029	0.0144	0.001
886	0.0136	0.0059	96.6588	-0.0044	0.0033	0.0137	-8.00E-04
887	0.0139	0.0072	96.7136	-0.0042	0.0027	0.014	-8.00E-04
888	0.0137	0.0055	96.7876	-0.0053	0.0034	0.0122	-0.001
889	0.0097	0.0049	96.8618	-0.0048	0.0038	0.0132	-0.0011
890	0.0076	0.005	96.9435	-0.0042	0.0045	0.0134	-0.0011
891	0.0034	0.0056	96.9982	-0.0058	0.0036	0.0128	-9.00E-04
892	0.0026	0.0061	97.0291	-0.0045	0.0039	0.0104	-0.0022
893	0.0015	0.0046	97.0478	-0.005	0.0057	0.0125	-8.00E-04
894	0.0041	0.0056	97.0466	-0.0049	0.0044	0.0102	-3.00E-04
895	0.0026	0.0051	97.0178	-0.0056	0.0055	0.0122	-0.0023
896	0.0027	0.0035	96.9671	-0.0033	0.0053	0.0113	-0.0017
897	0.0024	0.0038	96.921	-0.0039	0.0053	0.0107	-0.0015
898	0.0024	0.0046	96.8686	-0.0042	0.0049	0.0112	-0.0018
899	0.0028	0.0048	96.8218	-0.0064	0.0053	0.0129	-0.001
900	0.003	0.0046	96.7752	-0.0045	0.0048	0.0118	-0.0014
901	0.0048	0.0025	96.721	-0.004	0.0049	0.0114	-0.0012
902	0.0021	0.005	96.6858	-0.0055	0.0045	0.0123	-4.00E-04
903	0.0032	0.0062	96.6525	-0.0048	0.0058	0.0135	-0.0015

904	0.0051	0.0071	96.6237	-0.0046	0.0038	0.0132	-0.002
905	0.0041	0.0045	96.6081	-0.0036	0.0039	0.0132	-0.0019
906	0.0052	0.0063	96.6273	-0.0036	0.0035	0.0122	-0.0012
907	0.0039	0.0068	96.6587	-0.0043	0.0039	0.0121	-0.0021
908	0.0039	0.0077	96.7151	-0.0051	0.0034	0.0114	-0.0012
909	0.0034	0.0051	96.7649	-0.0047	0.0038	0.013	-0.0014
910	0.0058	0.0048	96.7999	-0.0052	0.0034	0.013	-0.0015
911	0.005	0.0066	96.8149	-0.0045	0.0035	0.0124	-4.00E-04
912	0.0055	0.0085	96.8059	-0.0043	0.0024	0.0129	-0.0034
913	0.0058	0.0056	96.8135	-0.0041	0.0023	0.0126	-0.0015
914	0.0078	0.0072	96.8204	-0.0039	0.0038	0.0128	-7.00E-04
915	0.0084	0.0065	96.8423	-0.0046	0.0036	0.0135	-0.002
916	0.0083	0.005	96.827	-0.0041	0.0047	0.0133	-0.0015
917	0.0091	0.0029	96.7481	-0.0037	0.0035	0.0141	-0.002
918	0.007	0.0072	96.6176	-0.0029	0.0029	0.0121	-0.0023
919	0.0061	0.004	96.3725	-0.0033	0.0031	0.0133	-0.0018
920	0.0051	0.004	95.7687	-0.0034	0.0038	0.0121	-0.0017
921	0.0058	0.0054	93.6137	-0.0039	0.0028	0.0126	-0.0014
922	0.0045	0.0075	88.767	-0.0033	0.0039	0.0114	-0.0017
923	0.005	0.0053	79.089	-0.0039	0.0046	0.0137	-0.0015
924	0.0055	0.0045	65.5372	-0.0042	0.003	0.0105	-0.001
925	0.0055	0.0057	46.948	-0.0039	0.0042	0.0135	-0.0027
926	0.0076	0.0039	30.4891	-0.004	0.0044	0.0132	-0.0017
927	0.0067	0.0038	17.6232	-0.0027	0.0037	0.0132	-7.00E-04
928	0.0069	0.006	8.4535	-0.0031	0.0042	0.0132	-0.0017
929	0.0068	0.0067	3.8754	-0.0032	0.0043	0.0127	-5.00E-04
930	0.0057	0.0058	1.8006	-0.0033	0.0045	0.014	-0.0013
931	0.0071	0.0037	0.913	-0.0029	0.0052	0.0124	-0.0017
932	0.0045	0.0044	0.5035	-0.0027	0.0053	0.0139	-0.0013
933	0.0065	0.0068	0.2837	-0.0031	0.0042	0.0124	-0.0015
934	0.0056	0.0058	0.1721	-0.0016	0.0062	0.0136	-9.00E-04
935	0.0076	0.0046	0.1119	-0.0028	0.0059	0.0119	-0.002
936	0.0063	0.0042	0.0796	-0.0019	0.0064	0.0133	-0.0013
937	0.0057	0.0057	0.0602	-0.002	0.0066	0.0119	-0.0015
938	0.0059	0.0067	0.0471	-0.0027	0.0062	0.013	-0.0015
939	0.0048	0.0049	0.0376	-0.0019	0.0073	0.0128	-0.001
940	0.0058	0.0045	0.0305	-0.0013	0.0072	0.0131	0
941	0.005	0.0049	0.0276	-0.001	0.007	0.0138	-0.0012
942	0.0045	0.0077	0.0245	-0.0017	0.0077	0.0118	-0.0011
943	0.0046	0.0059	0.023	3.00E-04	0.0073	0.0139	-0.0015
944	0.0049	0.005	0.022	-1.00E-04	0.0084	0.0126	-0.0012
945	0.0037	0.0038	0.0223	5.00E-04	0.0086	0.0141	-0.001
946	0.0028	0.0059	0.0202	0.0012	0.0089	0.0137	-9.00E-04
947	0.0045	0.007	0.0181	6.00E-04	0.0097	0.0138	-6.00E-04

948	0.0046	0.005	0.0166	8.00E-04	0.0113	0.0127	-6.00E-04
949	0.0057	0.0058	0.0168	0.0023	0.0114	0.0136	-0.0023
950	0.011	0.0057	0.0165	0.003	0.0117	0.013	-0.0019
951	0.0174	0.005	0.0143	0.0044	0.0121	0.014	-6.00E-04
952	0.0186	0.0064	0.0153	0.0064	0.0114	0.0139	-0.0017
953	0.0184	0.0062	0.0153	0.0088	0.0142	0.0122	-2.00E-04
954	0.0139	0.0048	0.0146	0.0125	0.0149	0.0139	-1.00E-04
955	0.0093	0.0068	0.0129	0.0199	0.0158	0.0126	-0.0019
956	0.0049	0.0047	0.0116	0.0305	0.0169	0.0141	-0.0011
957	0.003	0.0049	0.0117	0.0493	0.0188	0.0129	-8.00E-04
958	0.0032	0.0063	0.0105	0.0848	0.0197	0.0145	-1.00E-04
959	0.0033	0.0043	0.0123	0.151	0.0207	0.0133	1.00E-04
960	0.003	0.0065	0.01	0.287	0.0231	0.0144	-4.00E-04
961	0.0029	0.0067	0.0096	0.602	0.024	0.013	0.0012
962	0.0033	0.0038	0.0108	1.4208	0.0276	0.0142	9.00E-04
963	0.0024	0.004	0.012	3.1375	0.0298	0.014	0.0014
964	0.0024	0.0056	0.0109	7.2126	0.0325	0.0142	0.0034
965	0.0036	0.0046	0.0112	13.3984	0.0354	0.0152	0.0038
966	0.003	0.0044	0.011	22.4301	0.039	0.0148	0.0047
967	0.0032	0.0054	0.0119	33.0196	0.0446	0.0149	0.0059
968	0.0031	0.0042	0.0116	40.7161	0.0487	0.0157	0.0064
969	0.0028	0.0062	0.0107	46.8288	0.0548	0.0157	0.0064
970	0.0014	0.0054	0.0089	49.9896	0.0617	0.0164	0.0058
971	0.0036	0.0069	0.0108	50.0896	0.069	0.0162	0.0058
972	0.003	0.0071	0.0115	48.3728	0.0779	0.0169	0.0057
973	0.0051	0.0057	0.0105	45.1117	0.0886	0.0185	0.0064
974	0.0096	0.0069	0.0106	38.0501	0.1026	0.0195	0.0064
975	0.0158	0.0075	0.0109	29.6362	0.1186	0.0185	0.0061
976	0.0245	0.0057	0.0101	20.2696	0.1368	0.0198	0.0073
977	0.0261	0.0044	0.0088	11.9811	0.1606	0.0205	0.0071
978	0.0222	0.0054	0.0105	6.1731	0.1889	0.0209	0.0071
979	0.0143	0.0056	0.0111	2.4571	0.228	0.0229	0.0074
980	0.0085	0.0059	0.0108	1.065	0.2703	0.0216	0.0093
981	0.0029	0.0065	0.0095	0.4917	0.3258	0.0234	0.0073
982	0.0043	0.007	0.0105	0.2284	0.3999	0.0258	0.0077
983	0.0039	0.0056	0.0107	0.126	0.4972	0.027	0.0089
984	0.0022	0.0055	0.0105	0.0711	0.6301	0.0306	0.0084
985	0.0035	0.0071	0.0088	0.0418	0.8005	0.0337	0.0094
986	0.0041	0.0081	0.0082	0.027	1.0579	0.0412	0.009
987	0.0031	0.0076	0.0089	0.0175	1.4288	0.0523	0.0087
988	0.0021	0.0064	0.0075	0.012	1.916	0.07	0.0098
989	0.0022	0.0091	0.0095	0.0083	2.6414	0.1015	0.011
990	0.0024	0.0097	0.0071	0.0051	3.8116	0.1533	0.01
991	0.0022	0.0114	0.0075	0.0041	5.5773	0.2586	0.0115

992	0.0032	0.0114	0.009	0.004	8.2275	0.4578	0.0114
993	0.0033	0.0103	0.01	0.0019	12.6408	0.9216	0.0114
994	0.0029	0.0115	0.009	0.0017	18.8091	1.9944	0.0122
995	0.0031	0.0131	0.0093	3.00E-04	28.0201	4.4426	0.0128
996	0.0036	0.0124	0.0071	0	39.4728	9.8062	0.014
997	0.003	0.0117	0.0093	-2.00E-04	50.9455	18.6702	0.0143
998	0.003	0.0106	0.0076	-1.00E-04	63.2079	34.4702	0.015
999	0.0024	0.01	0.007	1.00E-04	72.3011	53.1611	0.0164
1000	0.0036	0.0071	0.0076	-7.00E-04	77.2098	71.2519	0.0166
1001	0.0043	0.0069	0.0099	-0.002	76.876	84.4383	0.0177
1002	0.0043	0.0079	0.0095	-0.0013	72.0574	92.5733	0.0185
1003	0.0051	0.0056	0.0087	-0.0017	61.6401	95.8639	0.0206
1004	0.0055	0.0064	0.009	-0.0019	51.1237	96.5632	0.0214
1005	0.0062	0.0057	0.0093	-9.00E-04	38.2021	96.8672	0.0229
1006	0.0058	0.0051	0.0079	-0.0025	28.4399	97.2569	0.0246
1007	0.0052	0.007	0.0099	-0.0027	18.9361	97.5463	0.0265
1008	0.0072	0.006	0.0079	-0.0032	12.8839	97.7247	0.0288
1009	0.0081	0.0058	0.0082	-0.0029	8.9644	97.7784	0.0308
1010	0.0112	0.0066	0.0091	-0.0029	5.8633	97.8308	0.0349
1011	0.0152	0.0048	0.0078	-0.0022	4.217	98.0606	0.0369
1012	0.0194	0.0074	0.0086	-0.0035	2.9637	98.2531	0.0397
1013	0.0216	0.0061	0.0084	-0.0032	2.1512	98.4481	0.0433
1014	0.0235	0.0056	0.01	-0.0031	1.599	98.5567	0.0476
1015	0.0222	0.0078	0.0107	-0.0025	1.1844	98.5655	0.0523
1016	0.0204	0.008	0.0108	-0.003	0.9419	98.4696	0.0595
1017	0.0171	0.0059	0.0115	-0.0032	0.7483	98.3728	0.0657
1018	0.0148	0.0063	0.01	-0.0047	0.5904	98.347	0.0731
1019	0.0132	0.0056	0.0099	-0.002	0.4855	98.316	0.0835
1020	0.011	0.009	0.0104	-0.0037	0.3997	98.4417	0.0931
1021	0.0104	0.0079	0.0082	-0.0043	0.3317	98.6547	0.1056
1022	0.0077	0.0078	0.0097	-0.0041	0.2748	98.8444	0.1222
1023	0.0046	0.0085	0.0088	-0.0037	0.2313	99.0093	0.1413
1024	0.0036	0.0081	0.0087	-0.0034	0.1967	99.0608	0.1629
1025	0.0043	0.0078	0.0099	-0.0027	0.1702	99.0485	0.1905
1026	0.0035	0.0115	0.0096	-0.0047	0.1485	98.9026	0.2208
1027	0.0035	0.009	0.0096	-0.0036	0.1308	98.748	0.2595
1028	0.0039	0.0136	0.0094	-0.0041	0.1142	98.6858	0.3116
1029	0.0036	0.0149	0.0076	-0.0045	0.1019	98.6872	0.3744
1030	0.0042	0.0157	0.0069	-0.0047	0.0905	98.8378	0.4562
1031	0.0051	0.0192	0.0088	-0.0048	0.0782	99.0359	0.5781
1032	0.0047	0.0187	0.0083	-0.0031	0.0722	99.2696	0.7025
1033	0.0056	0.0163	0.0098	-0.0039	0.0628	99.4066	0.9013
1034	0.0052	0.0144	0.0112	-0.0037	0.059	99.4946	1.152
1035	0.0075	0.0139	0.01	-0.004	0.0523	99.4512	1.5209

1036	0.0082	0.0096	0.0083	-0.004	0.0483	99.3217	2.0307
1037	0.011	0.0091	0.0099	-0.0041	0.0445	99.2815	2.6982
1038	0.012	0.0081	0.0114	-0.0056	0.0402	99.2599	3.8438
1039	0.0091	0.0073	0.0119	-0.0041	0.0369	99.2673	5.3427
1040	0.0118	0.0091	0.0115	-0.0046	0.0331	99.3481	7.7938
1041	0.0094	0.0056	0.0117	-0.0056	0.0317	99.5042	11.127
1042	0.0086	0.0083	0.0095	-0.0069	0.0275	99.4661	16.5304
1043	0.0103	0.0082	0.0126	-0.0091	0.0278	99.3015	23.671
1044	0.011	0.0082	0.0133	-0.0079	0.024	98.904	32.7974
1045	0.0098	0.0101	0.017	-0.0075	0.0224	97.6651	43.0063
1046	0.0078	0.0084	0.015	-0.0084	0.0214	94.2999	53.8246
1047	0.008	0.012	0.0146	-0.0084	0.0197	87.5597	63.9655
1048	0.0093	0.0087	0.0139	-0.0083	0.0189	76.7609	71.6379
1049	0.0076	0.0087	0.0156	-0.0077	0.0181	60.9778	76.01
1050	0.0086	0.0124	0.017	-0.0081	0.016	43.9177	76.1467
1051	0.0107	0.0097	0.0173	-0.0074	0.0153	28.5258	71.5369
1052	0.0098	0.0125	0.0168	-0.0103	0.0146	16.7121	62.947
1053	0.0108	0.0115	0.0173	-0.0093	0.0139	8.9456	51.4568
1054	0.0088	0.0135	0.018	-0.0092	0.0132	4.9608	40.3897
1055	0.0101	0.0099	0.0189	-0.0097	0.0103	2.7855	29.5306
1056	0.0118	0.0123	0.022	-0.0113	0.0101	1.5446	20.3721
1057	0.0098	0.0093	0.0186	-0.0114	0.009	0.9369	13.8988
1058	0.0099	0.0116	0.0181	-0.0128	0.0081	0.5724	9.1951
1059	0.0115	0.0105	0.0187	-0.0129	0.0057	0.3816	6.3956
1060	0.0088	0.0102	0.0201	-0.014	0.0081	0.27	4.484
1061	0.01	0.0126	0.0248	-0.0157	0.0061	0.1962	3.1183
1062	0.0079	0.009	0.023	-0.0167	0.0061	0.156	2.3439
1063	0.0058	0.0099	0.0218	-0.0179	0.0042	0.1025	1.7608
1064	0.0081	0.0074	0.0199	-0.0447	-0.0243	0.1155	1.2907
1065	-0.0239	-0.0248	-0.0141	-0.0188	0.0019	0.1056	1.0212
1066	0.0097	0.0081	0.0225	-0.0172	-0.0024	0.0947	0.829
1067	0.0026	0.004	0.0135	-0.0183	-0.003	0.0893	0.6515
1068	0.0032	0.0015	0.0166	-0.0188	-0.003	0.0859	0.5305
1069	0.0033	0.003	0.0153	-0.022	-0.0022	0.0852	0.4241
1070	0.0024	0.0048	0.0183	-0.0206	-0.0051	0.0837	0.3583
1071	0.0041	0.0032	0.0164	-0.0195	-0.0041	0.0854	0.303
1072	0.0032	0.0065	0.0186	-0.022	-0.0025	0.0858	0.2544
1073	0.0065	0.0075	0.0211	-0.0213	-0.0047	0.0874	0.2204
1074	0.0041	0.005	0.0246	-0.0181	-0.0044	0.0885	0.1892
1075	0.0078	0.0098	0.0229	-0.021	-0.007	0.0932	0.161
1076	0.0069	0.0041	0.0247	-0.0183	-0.005	0.0938	0.1387
1077	0.0068	0.007	0.0266	-0.0159	-0.0056	0.1013	0.1223
1078	0.0062	0.0099	0.031	-0.0195	-0.0066	0.098	0.1058
1079	0.0098	0.0089	0.0306	-0.0226	-0.0076	0.102	0.0891

1080	0.0104	0.0109	0.0366	-0.0256	-0.0113	0.102	0.0775
1081	0.0129	0.01	0.0364	-0.0278	-0.0076	0.1101	0.0659
1082	0.0157	0.013	0.0405	-0.0296	-0.0079	0.1111	0.058
1083	0.0182	0.0121	0.0446	-0.0297	-0.0072	0.117	0.0526
1084	0.0168	0.0147	0.0477	-0.0315	-0.0089	0.1216	0.0453
1085	0.0192	0.019	0.0488	-0.0267	-0.0078	0.1272	0.0387
1086	0.0172	0.0156	0.0491	-0.0216	-0.0128	0.1355	0.0341
1087	0.0217	0.0248	0.0559	-0.0254	-0.0064	0.1383	0.0314
1088	0.0223	0.0247	0.063	-0.0272	-0.009	0.1478	0.0259
1089	0.0259	0.0238	0.0629	-0.0289	-0.0013	0.1587	0.023
1090	0.0226	0.0281	0.0658	-0.0279	-7.00E-04	0.1674	0.0214
1091	0.0258	0.0298	0.0736	-0.0324	-0.0088	0.1751	0.0158
1092	0.031	0.0278	0.0757	-0.028	0.002	0.1669	0.0155
1093	0.0316	0.0332	0.0856	-0.0294	-5.00E-04	0.1756	0.0125
1094	0.0354	0.0406	0.0863	-0.0301	-3.00E-04	0.1846	0.0088
1095	0.0396	0.0392	0.0948	-0.028	0.0018	0.1927	0.0089
1096	0.0398	0.0507	0.1004	-0.034	0.0033	0.2079	0.0094
1097	0.0424	0.0538	0.1097	-0.0334	0.0066	0.2202	0.0026
1098	0.049	0.0513	0.1225	-0.0321	0.0109	0.2358	0.0081
1099	0.054	0.0539	0.1284	-0.0283	0.015	0.254	0.0038
1100	0.0556	0.056	0.1359	-0.0341	0.0181	0.2708	0.004

Fig. 3

Air exposure (min)	Lateral photocurrent (A)						
	CWL=700 nm	CWL=800 nm	CWL=900 nm	CWL=970 nm	CWL=1000 nm	CWL=1025 nm	CWL=1050 nm
5	8.07E-07	7.25E-07	1.28E-06	4.37E-08	8.84E-08	2.05E-07	1.27E-08
15	7.99E-07	7.21E-07	1.27E-06	4.29E-08	8.87E-08	2.04E-07	1.26E-08
25	7.74E-07	6.95E-07	1.22E-06	4.17E-08	8.78E-08	2.03E-07	1.21E-08
35	7.56E-07	6.45E-07	1.20E-06	4.09E-08	8.74E-08	1.97E-07	1.17E-08
50	7.47E-07	6.39E-07	1.19E-06	3.95E-08	8.29E-08	1.97E-07	1.16E-08

Fig. 4(b)

Voltage (V)	Current density (mA/cm^2)
-0.02245	-34.48452
0.00408	-34.19073
0.03061	-34.19454
0.05714	-34.26389
0.08367	-34.11923
0.1102	-34.32956
0.13673	-34.16813
0.16327	-34.20585
0.1898	-34.12831
0.21633	-34.10864
0.24286	-34.20534
0.26939	-34.11731
0.29592	-34.17906
0.32245	-33.93019
0.34898	-33.91447
0.37551	-33.86806
0.40204	-33.8355
0.42857	-33.6608
0.4551	-33.55424
0.48163	-33.27852
0.50816	-32.9618
0.53469	-32.20185
0.56122	-30.75733
0.58776	-28.23485
0.61429	-23.38715
0.64082	-15.6468
0.66735	-3.4486
0.69388	13.99855
0.72041	36.64452
0.74694	64.57546
0.77347	97.12119
0.8	134.05487
